# Automatic Segmentation of Pancreatic Tumors Using Deep Learning on a Video Image of Contrast-Enhanced Endoscopic Ultrasound

**DOI:** 10.3390/jcm10163589

**Published:** 2021-08-15

**Authors:** Yuhei Iwasa, Takuji Iwashita, Yuji Takeuchi, Hironao Ichikawa, Naoki Mita, Shinya Uemura, Masahito Shimizu, Yu-Ting Kuo, Hsiu-Po Wang, Takeshi Hara

**Affiliations:** 1 First Department of Internal Medicine, Gifu University Hospital, Gifu 501-1194, Japan; festinalenteyu@gmail.com (Y.I.); ichi.hiro.m.0814@gmail.com (H.I.); mitanao8@yahoo.co.jp (N.M.); ueshin550621@gmail.com (S.U.); shimim@gifu-u.ac.jp (M.S.); 2 Department of Electrical, Electronic and Computer Engineering, Faculty of Engineering, Gifu University, Gifu 501-1193, Japan; ytake@fjt.info.gifu-u.ac.jp (Y.T.); takeshi.hara@mac.com (T.H.); 3 Department of Integrated Diagnostics & Therapeutics, National Taiwan University Hospital, National Taiwan University College of Medicine, Taipei 10048, Taiwan; sfstruck@gmail.com; 4 Department of Internal Medicine, National Taiwan University Hospital, National Taiwan University College of Medicine, Taipei 10048, Taiwan; wanghp@ntu.edu.tw; 5 Center for Healthcare Information Technology (C-HIT), Tokai National Higher Education and Research System, Aichi 466-8560, Japan

**Keywords:** artificial intelligence, pancreatic cancer, endoscopic ultrasound, microbubble, contrast enhanced

## Abstract

Background: Contrast-enhanced endoscopic ultrasound (CE-EUS) is useful for the differentiation of pancreatic tumors. Using deep learning for the segmentation and classification of pancreatic tumors might further improve the diagnostic capability of CE-EUS. Aims: The aim of this study was to evaluate the capability of deep learning for the automatic segmentation of pancreatic tumors on CE-EUS video images and possible factors affecting the automatic segmentation. Methods: This retrospective study included 100 patients who underwent CE-EUS for pancreatic tumors. The CE-EUS video images were converted from the originals to 90-s segments with six frames per second. Manual segmentation of pancreatic tumors from B-mode images was performed as ground truth. Automatic segmentation was performed using U-Net with 100 epochs and was evaluated with 4-fold cross-validation. The degree of respiratory movement (RM) and tumor boundary (TB) were divided into 3-degree intervals in each patient and evaluated as possible factors affecting the segmentation. The concordance rate was calculated using the intersection over union (IoU). Results: The median IoU of all cases was 0.77. The median IoUs in TB-1 (clear around), TB-2, and TB-3 (unclear more than half) were 0.80, 0.76, and 0.69, respectively. The IoU for TB-1 was significantly higher than that of TB-3 (*p* < 0.01). However, there was no significant difference between the degrees of RM. Conclusions: Automatic segmentation of pancreatic tumors using U-Net on CE-EUS video images showed a decent concordance rate. The concordance rate was lowered by an unclear TB but was not affected by RM.

## 1. Backgrounds

Treatment strategies differ greatly between malignant pancreatic tumors, such as those in pancreatic cancer and neuroendocrine, and benign tumors, such as those observed in autoimmune or tumor-forming pancreatitis. Therefore, differential diagnosis of these tumors is important for the appropriate selection of treatment. Cross-sectional imaging studies, including computed tomography (CT) and magnetic resonance imaging (MRI), are widely used for the detection and differentiation of pancreatic tumors and are reported to have relatively high diagnostic capabilities for determining malignancy [1]. However, false-positive results on these cross-sectional imaging studies can be a problem. For example, some research has reported that after imaging studies suggested malignancies, surgical resection of pancreatic tumors eventually revealed negative results for neoplastic disease [2]. Therefore, pathological evaluation is usually required for the accurate differential diagnosis of pancreatic tumors. Endoscopic ultrasound-guided fine-needle aspiration (EUS-FNA) has an important role in obtaining pathological material from pancreatic tumors because of its high diagnostic yields for malignancy [3,4,5,6]. The recent development of newly designed fine needle biopsy needles has further improved sample acquisition rates, with improved diagnostic accuracy [7,8,9,10,11,12]. While EUS-FNA is considered a safe procedure, it still has a possibility of false-negative results and the risk of adverse complications such as bleeding or pancreatitis due to needle puncture [13]. Moreover, needle tract seeding was reported in patients who underwent EUS-FNA for pancreatic cancer [14,15]. Ideally, the diagnosis should be made with more accurate imaging studies that do not use needle puncture.

Endoscopic ultrasound (EUS) is an imaging technique that enables the evaluation of pancreatic lesions with high spatial resolution images from the upper intestine by using a special endoscope equipped with a high-frequency transducer at the tip [16]. Recently, developments made to several techniques of image enhanced EUS have further improved the diagnostic accuracy [12,17]. Contrast-enhanced EUS (CE-EUS) is one technique of image enhanced EUS. CE-EUS is a novel imaging modality that detects signals from microbubbles in vessels and allows evaluation of real-time perfusion and microvasculature of pancreatic tumors [16,18,19,20,21,22]. The diagnostic sensitivity and specificity for pancreatic ductal carcinoma by qualitative evaluation of enhancement pattern during CE-EUS were reported as 95.1% (95% confidence interval (CI) 92.7–96.7%) and 89.0% (95% CI 83.0–93.1%), respectively [23]. However, the problem with the qualitative evaluation is that it is subjective, which may cause variability in the evaluation between endoscopists. To overcome this disadvantage, the time intensity curve (TIC), which is a chart showing the change over time of the echo intensity on the specified area inside the tumor, was introduced. TIC was used as an objective qualitative evaluation of CE-EUS and reportedly improves the diagnostic accuracy of pancreatic tumors [24]. The qualitative analysis using TIC showed that the sensitivity and specificity for differential diagnosis of pancreatic cancer from mass-forming pancreatitis were 93.8% and 89.5%, respectively [25]. However, TIC cannot be obtained appropriately for tumors that have intense respiratory movement (RM) on the EUS image because the measurement area is fixed on one part of the image plane. Therefore, the results of TIC may change depending on the selected area, especially in tumors with heterogeneous enhancement.

Recently, deep learning has been developed as a method that can automatically extract complex features from the raw image data [26]. Convolutional neural networks (CNNs) were introduced as an automated classification method for various images, and their use has been expanded to detect and segment background imaging [27,28]. U-Net was introduced as one of the CNNs in 2015, with state-of-the-art performance in medical image segmentation [29]. The automatic segmentation of pancreatic tumors on video images of CE-EUS using U-Net and deep learning of enhancement patterns over time in the segmented area for classification might improve the diagnostic accuracy and overcome the above-mentioned limitations of the qualitative and quantitative analyses of CE-EUS. We conducted this study to evaluate the capability of automatic segmentation for pancreatic tumors on the video images of CE-EUS using deep learning, specifically U-Net. Possible factors, such as RM or unclear tumor boundary (TB), that might have affected the automatic segmentation using deep learning were also evaluated.

## 2. Patients and Methods

### 2.1. Patients Selection

This retrospective study was conducted at two academic centers: National Taiwan University Hospital, Bei-Hu Branch, and Gifu University Hospital. The study took place between March 2016 and February 2019. CE-EUS video image sets of pancreatic tumors were created for training and testing of automatic segmentation using deep learning. Study participants met the following inclusion criteria: patients who underwent CE-EUS for pancreatic solid tumors and had a diagnosis based on either one of the reference methods; surgical diagnosis or fine-needle aspiration (FNA) diagnosis with a compatible clinical course with a follow-up period of more than 6 months. Patients who met the following criteria were excluded from the study: CE-EUS video image was less than 90 s after the injection of the contrast agent; the image was of poor quality, for example, displaying shadows caused by air. Through the patient selection process, 100 individuals were identified and included in this analysis. Informed consent was obtained in the form of an option to opt out. All procedures performed in the studies involving human participants were in accordance with the ethical standards of the institutional research committee at each center and were also in accordance with the 1964 Declaration of Helsinki as well as its later amendments and comparable ethical standards.

### 2.2. Contrast Enhanced-Endoscopic Ultrasound

**Figure 1:**
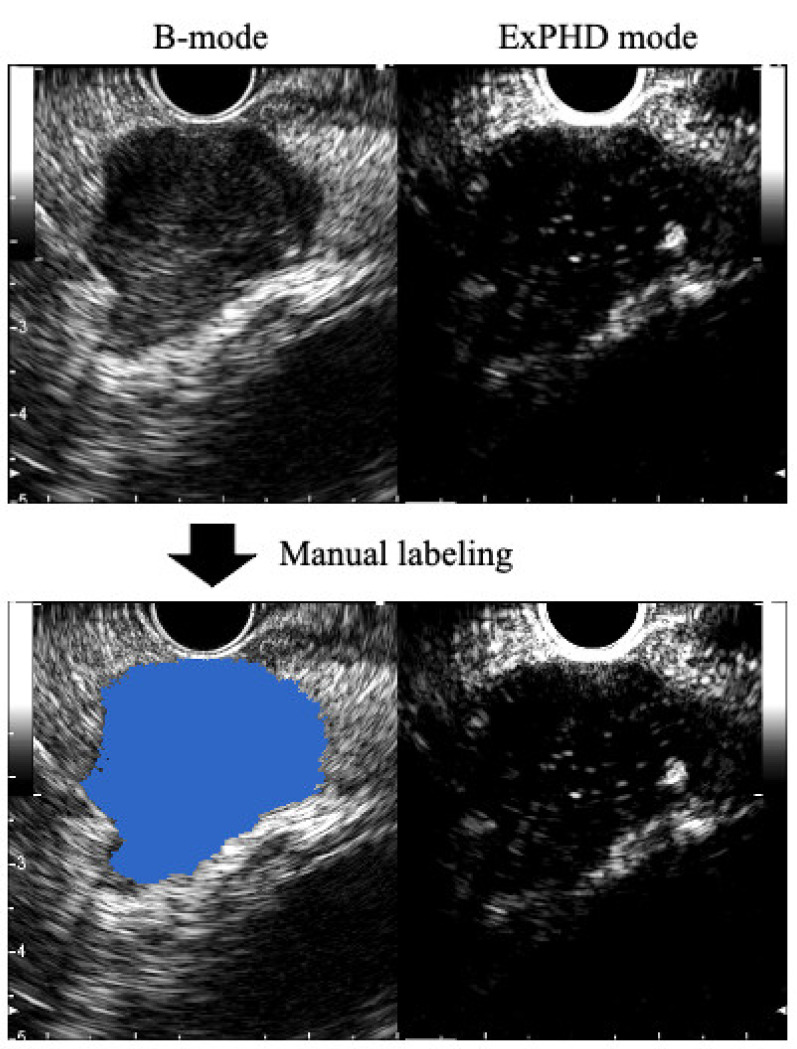
Manual labeling. Image of CE-EUS. The left side of the image is B-mode, and the right side is the extended pure harmonic detection (ExPHD) mode. The tumor was labeled with blue color as the ground truth on each image. CE-EUS: Contrast-enhanced endoscopic ultrasound.

CE-EUS was performed using a convex-type echoendoscope (GF-UCT260; Olympus, Tokyo, Japan) and ultrasound processors (ProSound F75 or ProSound α10; Hitachi-Aloka Medical, Tokyo, Japan, or EU-ME2; Olympus, Tokyo, Japan). The setting of the ultrasound processor was changed to dual imaging with the extended pure harmonic detection mode on the right side and standard B-mode on the left side (Figure 1). The gain and range levels were adjusted so that the entire tumor could be visualized with appropriate contrast. Microbubbles were composed of perfluorobutane, created to a median diameter of 2–3 μm (Sonazoid: Daiichi-Sankyo, Tokyo, Japan; GE Healthcare, Milwaukee, WI, USA), and were used as the ultrasound contrast agent. A video image of the screen was recorded while observing the pancreatic tumor for 90 seconds after intravenous administration of the contrast agent.

### 2.3. Preparation of the Training Data Sets

An original video image of CE-EUS in DICOM format with 30 frames per second (FPS) was converted to still images in RAW format so that one video image had approximately 900 still images per patient. The first of every five still images was selected, and these were then again converted to DICOM format with 6 FPS and used for all subsequent analyses. On each frame of the CE-EUS video, the pancreatic tumor was manually labeled on the B-mode image by two experienced endosonographers (Y. I. and T. I.) as the ground truth (Figure 1). The severity of RM was classified into three grades: RM-A, less than 50% of the tumor diameter; RM-B, 51–99% of the tumor diameter; and RM-C, more than 100% of the tumor diameter. The TB was also classified into three grades based on how much the boundary was visible all around: TB-1, visible all around; TB-2, visible 51–99% around; and TB-3, visible less than 50% around (Table 1).

### 2.4. Deep Learning and Automatic Segmentation

**Figure 2:**
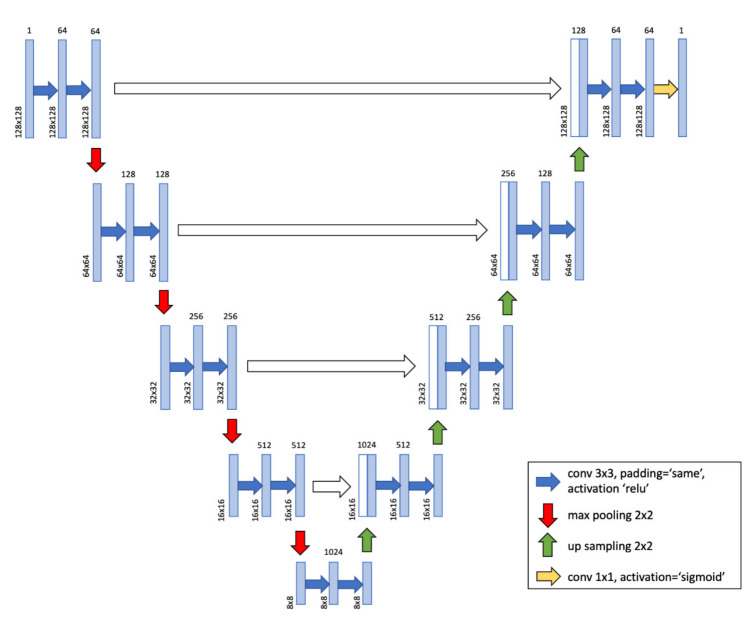
U-Net architecture. A deep learning network with U-net architecture was used for this study.

The U-Net architecture consists of a contracting path and an expansive path (Figure 2). The contracting path (the left side of “U”) is a down-sampling stage, which consists of several repetitions of two convolutions and one max-pooling layer with kernel sizes of 3 × 3 and 2 × 2, respectively. The expansive path (the right side of “U”) is an up-sampling stage, which consists of the repetition of the concatenation of the features extracted from corresponding layers in the contracting path (copy and crop), two convolutions, and one up-sampling (up-conv) layer. In this path, the kernel for the convolution layer is the same as that for the contracting path and 2 × 2 for the up-sampling layer. The ground truth data were used as the training data. Automatic segmentation was performed using U-Net for 100 epochs and was evaluated with 4-fold cross-validation.

### 2.5. Study Outcomes and Statistical Analysis

The primary endpoint was the concordance rate between the automatic segmentation area and the grand truth area of the video images in 4-fold cross-validation. The secondary endpoints were to evaluate the effect of RM and TB on the concordance rate.

**Figure 3:**
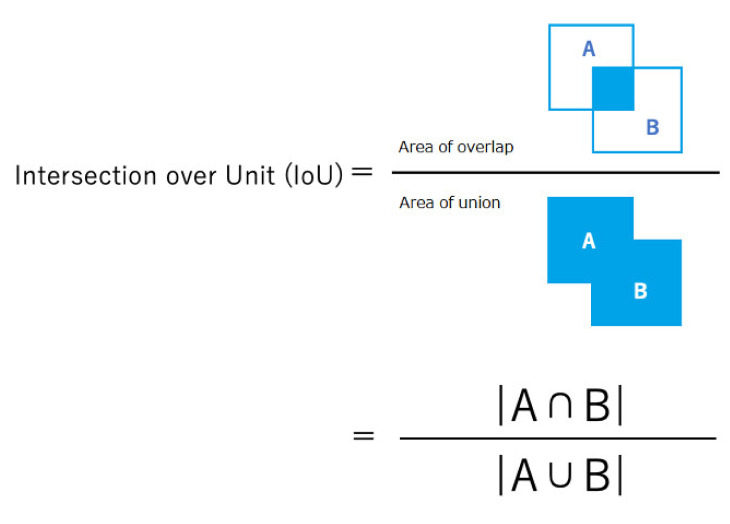
The Intersection over Unit (IoU). The formula of IoU. The IoU is the value between 0 and 1, calculated by dividing the area of overlap with the area of union.

The concordance rate between the grand truth area and the automatic segmentation area was calculated using the IoU, which is a value ranging from 0 to 1 that is calculated by dividing the area of overlap between the ground truth and the automatic segmentation area by the area of union (Figure 3). The IoU of each whole video image was calculated and presented as a median (range). The Mann–Whitney U-test was used to compare continuous variables. For categorical variables, comparisons were made using either the Chi-square test or the Fisher’s exact test, as appropriate. A *p*-value of less than 0.05 was considered statistically significant. All statistical analyses were performed using the statistical package R (version 3.5.2, Vienna, Austria).

## 3. Results

### 3.1. Characteristics of Patients

One hundred patients (52 men and 48 women; median age, 70 years; range, 29–89 years) were included in this study. The median diameter of the tumors was 24 mm (range, 8–91 mm). Tumors were located at the head of the pancreas in 37 patients and at the body/tail of the pancreas in 63 patients. The final diagnoses were pancreatic cancer in 67 patients, neuroendocrine tumor in 10 patients, autoimmune pancreatitis in 7 patients, metastatic pancreatic tumor in 6 patients, chronic pancreatitis in 4 patients, malignant lymphoma in 2 patients, solid pseudopapillary neoplasm in 2 patients, fat necrosis in 1 patient, and mass forming pancreatitis in 1 patient. The respiratory movement was classified as MR-A in 70 patients, RM-B in 19 patients, and RM-C in 11 patients. The tumor boundary was classified as TB-1 in 40 patients, TB-2 in 50 patients, and TB-3 in 10 patients. (Table 2).

### 3.2. The Concordance Rate between the Grand Truth Area and Automatic Segmentation Area

**Figure 4:**
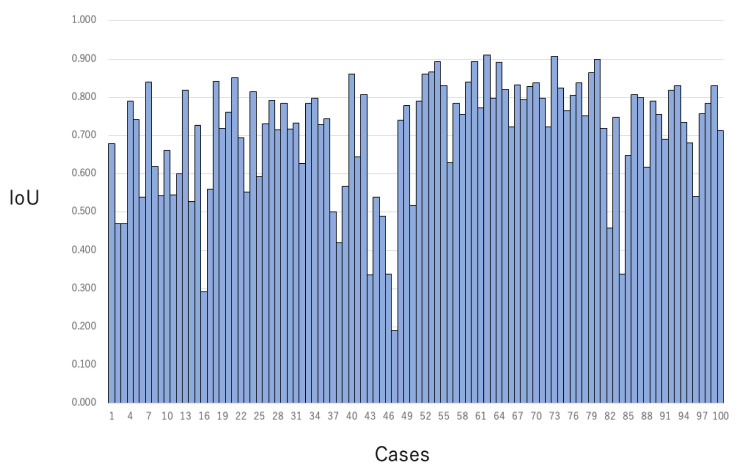
Intersection over Unit (IoU) of each case. IoUs of each case were shown.

**Figure 5:**
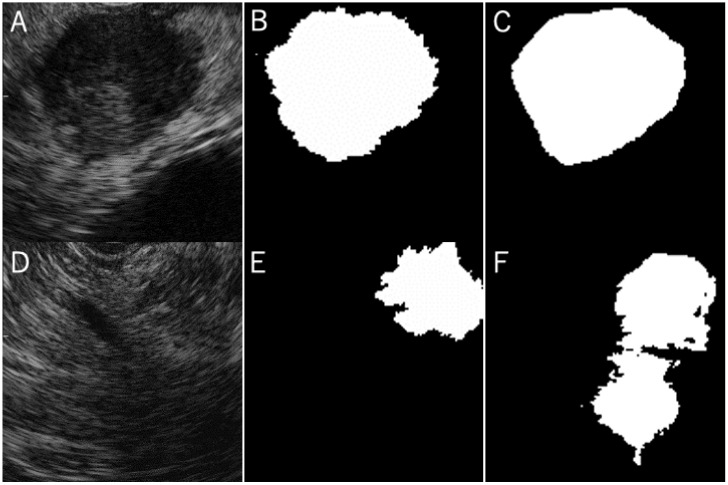
Cases of the highest and lowest concordance. Images of tumor in B-mode of highest intersection over unit (IoU) case (**A**). The manually labeled area (**B**) was almost the same as the automatic segmented area (**C**). Images of tumor in B-mode of lowest IoU case (**D**). The manually labeled area (**E**) was not similar to the automatic segmented area (**F**).

**Figure 6:**
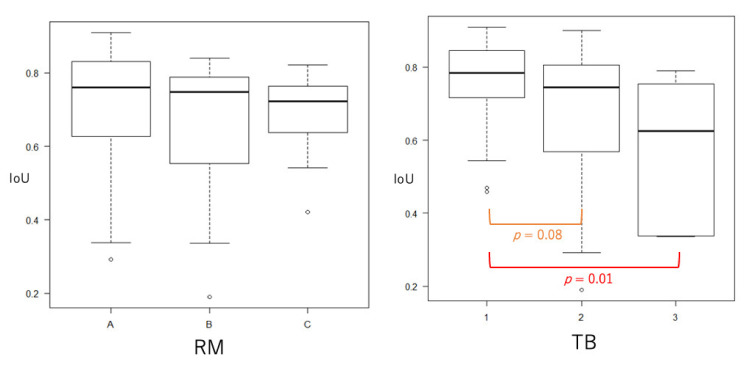
Intersection over Unit by the classification of respiratory movement and tumor boundary. No significant difference was seen in the intersection over unit (IoU) between the groups of RM; however, IoU in TB-1 was significantly higher than that in TB-3.

The median IoU of all cases was 0.77 (range, 0.39–0.91) (Table 3, Figure 4). The case with the highest IoU of 0.91 had RM-A and TB-1 (Figure 5, Video S1). The case with the lowest IoU of 0.13 had RM-B and TB-2 (Figure 5, Video S2). As for the tumor boundary classification, the median IoU in TB-1, TB-2, and TB-3 were 0.80, 0.76, and 0.69, respectively. The IoU in TB-1 was significantly higher than that in TB-3 (*p* < 0.01). However, the median IoU of respiratory movement classifications in RM-A, RM-B, and RM-C were 0.77, 0.79, and 0.76, respectively, and there was no significant difference between the degrees (Table 3, Figure 6).

## 4. Discussion

This study evaluated the capability of automatic segmentation for pancreatic tumors using U-Net as deep learning on the video image of CE-EUS. There have been a number of reports regarding the segmentation of specific subjects using deep learning on ultrasound images. Anas et al. evaluated the automatic segmentation using deep learning for the prostate on still images during a trans-rectal ultrasound and showed a mean Dice similarity coefficient of 93% with a U-Net-based original convolutional network [30]. Another study by Hu et al. evaluated the automatic segmentation of breast tumors on still images of breast ultrasounds and showed a mean Dice similarity coefficient of 88.97% using a dilated fully convolutional network (DFCN) [31]. Li et al. evaluated the automatic segmentation of gastrointestinal stromal tumors in EUS images using a multi-task refined boundary-supervision U-net (MRBSU-Net) and showed a mean Dice similarity coefficient of 92% [32]. Although the used CNN and the subject were different between the studies, the concordance rates of these three studies were around 90% and were considered as favorable in comparison with that of our study (the median IoU of 0.77). Another possible cause of this difference could be that the three studies used still ultrasound images, which were usually taken with the clearest view of the subject lesion for automatic segmentation. However, the EUS video images used in the current study consisted of approximately 540 serial still images and possibly contained unclear views of the subject, as the respiratory movement and body motion of the patient made it difficult to maintain a clear view of the pancreatic tumor.

As a nature of ultrasound examination, ultrasound images can have a significant amount of artifact from the structure inside or around the subjective lesion since it is difficult for the ultrasound wave to penetrate air, bone, or calcification. Furthermore, during visualization of the pancreatic tumor on EUS, EUS images can be affected by calcification of the tumor or pancreatic parenchyma or by presenting air around the transducer at the tip of the EUS. These artifacts make the boundary of the pancreatic tumor on the EUS image unclear, which may complicate the automatic segmentation of the pancreatic tumor using deep leering. Respiratory movement during observation, which caused the displacement of the lesion on the video images, might also interrupt the accurate segmentation of the pancreatic tumor. Therefore, the effect of the degree of respiratory movement and unclear boundary on the concordance rate was evaluated in this study. Our results showed no significant difference in concordance rates between the degrees of respiratory movement; however, there were significant differences in the concordance rates in the degree of the unclear tumor boundary. The TB1 group (the boundary of visibility all around) showed a significantly higher concordance rate than the groups of TB2 (tumor boundary of visibility 51–99%) or TB3 (tumor boundary of visibility less than 50%). Therefore, it might be better to include only pancreatic tumors with clear boundaries on the EUS video image for automatic segmentation using U-Net, if a higher concordance rate is required for subsequent analysis.

Different or novel algorithms of deep learning might improve the segmentation capability of the subject lesion even on ultrasound imaging. In automatic tumor segmentation in breast ultrasound images, a study by Hu et al. compared six different algorithms—VGG-16 network, U-Net, dilated residual network, DFCN with and without dilated convolution, DFCN with a phase-based active contour model—that were fine-tuned from a pre-trained model and showed DFCN with a phase-based active contour model showed the highest Dice similarity coefficient [31]. As for EUS images, Li et al. compared five different algorithms—U-Net, RU-Net, multi-task RU-Net, refined boundary-supervision U-net (RBSU-Net), and MRBSU-Net—in the automatic segmentation of gastrointestinal stromal tumors, and MRBSU-Net showed the highest concordance [32]. However, which algorithm is better in automatic segmentation for a certain subject of EUS video image has been rarely reported and is a question for future research.

Once the accurate automatic segmentation of pancreatic tumors on B-mode video images of CE-EUS is achieved, the next step is deep learning of enhanced patterns of CE-EUS on the segmented area of the pancreatic tumor for differential diagnosis. Currently, the qualitative analysis of CE-EUS based on enhancement patterns, such as hypo or hyper and homogenous or heterogenous enhancement, was made based on the impression of operators, which might have lower reliability and validity because of subjective evaluation. For the quantitative analysis, which is an objective evaluation, TIC cannot be obtained appropriately for tumors having intense respiratory movement or heterogenic enhancement on the video image of CE-EUS because the measurement area is fixed on one part of the image plane. We then think the deep learning of enhancement pattern on the automatically segmented pancreatic tumor area on the video image of CE-EUS has overcome these current limitations of CE-EUS for pancreatic tumors and has a possibility to improve the diagnostic accuracy of CE-EUS for pancreatic tumors, even if the operators do not have much experience for that.

This study had several limitations. This study only evaluated the automatic segmentation capability, determining the boundaries and areas of the pancreatic tumors, on the EUS video images using U-Net and possible artifacts affecting the results; however, it is still unclear how much accuracy on segmentation is required for subsequent analysis, such as classification, since the automatic segmentation was the first step of the automatic differential diagnosis. Another limitation is the EUS video images retrospectively obtained at two academic centers that might decrease the external viridity of the study in terms of patient selection and CE-EUS itself. Finally, U-Net was the only algorithm used for the automatic segmentation in this study. Other algorithms should be evaluated to find the best algorithm for pancreatic tumor segmentation of the CE-EUS video image.

## 5. Conclusions

Automatic segmentation of pancreatic tumors using deep learning, specifically U-Net, on the video images of CE-EUS showed a decent concordance rate, with a median IoU of 0.77. The unclear boundary lowers the concordance rate, although the respiratory movement did not affect the rate. Further studies evaluating other algorithms for automatic segmentation are required to improve the concordance rate.

## Figures and Tables

**Table 1:** Classification of degree on respiratory movement and tumor boundary.

Respiratory Movement	
RM-A	Respiratory movement of less than 50% of the tumor diameter
RM-B	Respiratory movement of 51–99% of the tumor diameter
RM-C	Respiratory movement of more than 100% of the tumor diameter
Tumor boundary	
TB-1	Tumor boundary of visibility all around
TB-2	Tumor boundary of visibility 50–99% around
TB-3	Tumor boundary of visibility less than 50% around

RM: respiratory movement; TB: tumor boundary.

**Table 2:** Patient Characteristics.

Age, Years, Median (Range)	70 (29–89)
Gender, *n*, (male/female)	52/48
Tumor diameter, mm, median (range)	24 (8–91)
Tumor location, *n* (head/body or tail)	37/63
Final diagnosis, *n*	
Pancreatic cancer	67
Neuroendocrine tumor	10
Autoimmune pancreatitis	7
Metastatic pancreatic tumor	6
Chronic pancreatitis	4
Malignant lymphoma	2
Solid pseudopapillary neoplasm	2
Fat necrosis	1
Mass forming pancreatitis	1
Degree of respiratory movement, group, *n*	
RM-A	70
RM-B	19
RM-C	11
Degree of tumor boundary, group, *n*,	
TM-1	40
TM-2	50
TM-3	10

**Table 3:** The number of cases and Intersection over Unit for each class and overall.

*N* IoU, Median (Range)		TB Group			Total
		1	2	3	
RM group	A	34 0.80 (0.45–0.92)	33 0.77 (0.30–0.91)	3 0.61 (0.32–0.77)	70 0.78 (0.30–0.91)
	B	4 0.80 (0.79–0.81)	12 0.75 (0.13–0.85)	3 0.33 (0.32–0.80)	19 0.79 (0.13–0.85)
	C	2 0.76 (0.72–0.81)	5 0.79 (0.58–0.84)	4 0.76 (0.47–0.77)	11 0.76 (0.46–0.84)
total		40 0.80 (0.45–0.91)	50 0.76 (0.13–0.91)	10 0.68 (0.32–0.80)	100 0.77 (0.13–0.92)

IoU, Intersection over Unit.

## Data Availability

The data that support the findings of this study are available from the corresponding author upon reasonable request.

## Supplementary Materials

The following are available online at https://www.mdpi.com/article/10.3390/jcm10163589/s1, Video S1: automatic segmentation of pancreatic tumor with the highest intersection over unit; Video S2: automatic segmentation of pancreatic tumor with the lowest intersection over unit.
